# Membrane testosterone binding sites in prostate carcinoma as a potential new marker and therapeutic target: Study in paraffin tissue sections

**DOI:** 10.1186/1471-2407-5-148

**Published:** 2005-11-17

**Authors:** Constantina Dambaki, Christina Kogia, Marilena Kampa, Katherine Darivianaki, Michael Nomikos, Ploutarchos Anezinis, Panayiotis A Theodoropoulos, Elias Castanas, Efstathios N Stathopoulos

**Affiliations:** 1 Department of Pathology, University of Crete, School of Medicine, P.O. Box 2208, Heraklion, GR-71003, Crete, Greece; 2 Department of Experimental Endocrinology, University of Crete, School of Medicine, P.O. Box 2208, Heraklion, GR-71003, Crete, Greece; 3 Department of Urology, University of Crete, School of Medicine, P.O. Box 2208, Heraklion, GR-71003, Crete, Greece; 4 Department of Biochemistry, University of Crete, School of Medicine, P.O. Box 2208, Heraklion, GR-71003, Crete, Greece

## Abstract

**Background:**

Steroid action is mediated, in addition to classical intracellular receptors, by recently identified membrane sites, that generate rapid non-genomic effects. We have recently identified a membrane androgen receptor site on prostate carcinoma cells, mediating testosterone rapid effects on the cytoskeleton and secretion within minutes.

**Methods:**

The aim of this study was to investigate whether membrane androgen receptors are differentially expressed in prostate carcinomas, and their relationship to the tumor grade. We examined the expression of membrane androgen receptors in archival material of 109 prostate carcinomas and 103 benign prostate hyperplasias, using fluorescein-labeled BSA-coupled testosterone.

**Results:**

We report that membrane androgen receptors are preferentially expressed in prostate carcinomas, and they correlate to their grade using the Gleason's microscopic grading score system.

**Conclusion:**

We conclude that membrane androgen receptors may represent an index of tumor aggressiveness and possibly specific targets for new therapeutic regimens.

## Background

The biological activity of androgen occurs predominantly through binding to an intracellular androgen receptor (iAR) protein, a member of the nuclear receptor superfamily, functioning as a ligand-activated transcription factor [1]. However, in recent years, a number of reports indicate additional rapid androgen actions, including the rapid activation of kinase-signaling cascades, modification of actin cytoskeleton and modulation of intracellular calcium levels [see [2,3], for reviews]. These actions are considered to be non-genomic in nature because they occur in cells lacking functional iARs in the presence of inhibitors of transcription and translation, or are observed too rapidly to involve changes in gene transcription [4].

We have previously identified membrane androgen receptor (mAR) sites in prostate and breast cancer cell lines [5-7]. In a preliminary work performed on freshly prepared epithelial cells from prostate carcinoma, non-cancerous peritumoral tissue, and benign prostate hyperplasia (BPH), we have shown that mARs are expressed preferentially on carcinoma cells [8]. Activation of mARs induces actin cytoskeleton polymerization and redistribution [5,9], secretion of Prostate Specific Antigen (PSA) and apoptosis [6,7]. Membrane androgen receptor sites were responsible for the induction of testosterone-BSA-induced apoptosis in T47D breast cancer cells [7]. It is interesting to note that mARs may be different from iARs, as they are not recognized by antibodies against the latter [5], and are not inhibited by a series of commonly used antiandrogens in vitro [9] or in vivo [6].

In the present work we developed a method for the in situ detection of mARs in formalin-fixed and paraffin-embedded tissues. The aim of the present study was to: (1) Validate a method for the detection of mARs in formalin-fixed paraffin-embedded specimens of prostate tumors, and (2) explore the distribution of mARs in prostate carcinomas. More specifically, we have tried to reply to the following questions: (a) Are mARs equally distributed in cases of prostate carcinoma and BPH, and (b) are mARs expressed in prostate carcinoma related to the gravity of the disease, as expressed by the Gleason's score?

## Methods

One hundred and nine (109) cases of prostate carcinoma (age 45–93 years, mean ± SD 71.17 ± 9.02, median 71 years, followed for 4 to 72 months) and one hundred and three (103) cases of BPH (age 37–92, mean ± SD 70.44 ± 8.38, median 70 years), retrieved from the archives of the Pathology Department of the University Hospital of Crete, were analyzed retrospectively. Prostate carcinoma specimens corresponded to 23 radical prostatectomies, 15 transurethral resections harboring incidentally encountered carcinoma, and 71 transrectal biopsies. Three cases of carcinoma diagnosed in transurethral resection specimens were rejected for technical reasons. Survival of patients ranged from 1–72 months (mean ± SD 31.4 ± 9.32, median 29.5 months). Benign prostate hyperplasia cases corresponded to 96 transurethral resection and 7 prostatectomy specimens. Hematoxylin and Eosin (H&E) stained slides were reviewed by two investigators independently and blindly to the patients' clinical data. The Gleason's sum (combined score, Gleason's score) [10,11] of carcinoma cases was reevaluated by two observers. The results obtained from the histological study of carcinomas using the Gleason's grading system are presented in Table 1. In case of discrepancy between the two observers, the final decision was reached by consensus.

A set of three serial, 3 μm thick, tissue sections, embedded in Paramat extra (BDH Lab Supplies, Poole, UK), were taken on negatively charged slides (SuperFrost Plus, Kindler O GmbH, Freiburg, Germany) from each representative paraffin block. Two of the slides were used for the needs of mAR detection, while the third was stained with H&E for morphological study, and for grading in the case of carcinomas.

In order to evaluate the presence of mAR in tissue sections, we had to (partially) regenerate membrane proteins. In this aim, in an initial set of experiments, we have assayed different combinations of melting temperatures and times of incubation. Our findings indicated that the optimal conditions for the identification of the highest specific binding on the target molecules (mARs) were achieved by mild melting of the embedding medium (Paramat extra) at 42.5°C for 20 min, followed by dewaxing in xylene (three times, 10 min. each) and rehydration in decreasing concentrations of ethanol. Then, specimens were washed in distilled water for 20 min. and incubated at 37°C in a citrate buffer 0.01 M (pH 6.2) for two hours. Finally, they were washed in Tris Buffered Saline (TBS, 10 mM, NaCl 150 mM, pH 7.4) and processed for the detection of mARs. As in the present study we used albumin-conjugated steroids, and in order to minimize the non-specific binding of BSA we pre-incubated slides with 3% BSA for 40 min. Then the slides were washed in TBS and one of them (test slide) was incubated for 1 hour with 10^-6 ^M Testosterone-BSA-FITC while the other (control slide) with 10^-6 ^M BSA-FITC (both reagents were obtained from Sigma, St Louis, MO) in TBS.

Another potential source of non-specific staining could be the association of the ligand (testosterone) with iARs, due to the fact that microscopic tissue sections contain broken (sectioned) cells. Since our previous experiments have shown that classical antiandrogens react with iARs but not with mARs [6,9], we routinely used cyproterone acetate (a specific androgen receptor antagonist, Sigma, St Louis, MO) at a concentration of 10^-4 ^M, diluted in the testosterone-BSA-FITC or BSA-FITC solution in order to block iARs (see Results). After one-hour incubation, slides were rinsed with TBS, coverslipped using Polyvinyl Alcohol Mounting Medium with Dabco Antifading (Fluka Biochemika) and examined under a fluorescence microscope.

An introductory comparative study using both a confocal laser scanning microscope (Leica TCS SP) and a fluorescence microscope (Nikon Microphot-FXA fluorescence microscope, mounted with a Nikon DX-DB2 photographic camera) showed no significant advantage of the confocal over the conventional fluorescence microscope. The latter was therefore used for the routine detection of mARs. Results were expressed as a percentage of mAR positive cells in reference to total number of cells examined in 10 sequential high power fields (X400) in a representative area of each test slide. The localization and pattern of staining were recorded.

Clinical data, namely age, previous treatment, follow-up and survival, were retrieved from the patients' files. Statistical analysis was performed by the Systat V 10.0 (SPSS, Chicago, IL) program. In the case of positive/negative cases of BPH and prostate carcinoma, we have used significance limits of the four-fold table test, using the χ^2 ^test, with 1 degree of freedom. The same test was used for the comparison of positive and negative cases in each group. For exploring the correlation between positive/negative cases of mAR expression and other parameters (age, treatment, survival, Gleason's sum) the Spearman rank correlation coefficient was calculated. The Mann-Whitney U test was used to compare the distribution of continuously-scaled outcomes by mAR expression. All statistical evaluations were double-sided.

This study was approved by the Ethics Committee of the University Hospital of Heraklion, and the Ethics Committee of the University of Crete.

## Results

### 1. Validation of the method

Previous results indicated that mARs can be detected in freshly prepared prostate carcinoma tissue [8]. The method presented here for the *in situ *detection of mARs is based on the binding of a ligand (FITC-BSA-bound testosterone) on mARs. Membrane androgen receptors are membrane proteins [5,12], expected to be (partially) denatured in microscopic tissue sections by formalin fixation, paraffin embedding and high temperatures, conditions known to modify integral membrane proteins. Therefore, we attempted to renaturate these proteins by incubating the tissue sections at relatively low temperatures during deparaffinization and renaturation process (mild tissue dewaxing and protein renaturation). Different combinations of melting temperatures (from 40° to 60°C), incubation times, xylene treatment and acidification were tried. As indicated in the Methods section, the best results were obtained with a mild paraffin melting and protein renaturing temperature of 42.5°C, followed by mild acidification in citrate buffer to dissociate any ligands loosely-bound to mARs or iARs [13] and extensive washing with TBS pH 7.4.

**Figure 1:**
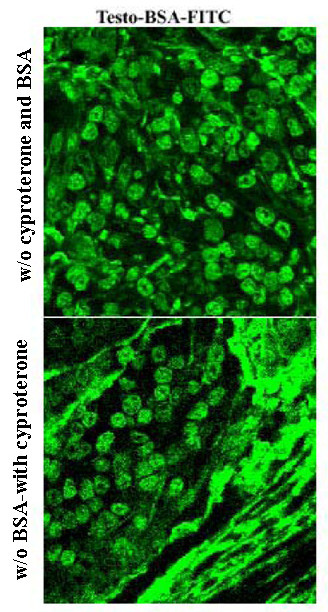
*Prostate carcinoma staining with testosterone-BSA-FITC*. Representative patterns of prostate carcinoma tissue section staining with testosterone-BSA-FITC in absence of BSA and cyproterone acetate preincubation (upper panel) or in presence of cyproterone acetate and in absence of BSA (lower panel). As explained in the text, BSA preincubation decreases or eliminates non-specific interactions of the tracer (testosterone-BSA-FITC) with prostate stroma or cell membranes, while the antiandrogen cyproterone acetate binds selectively to iAR.

**Figure 2:**
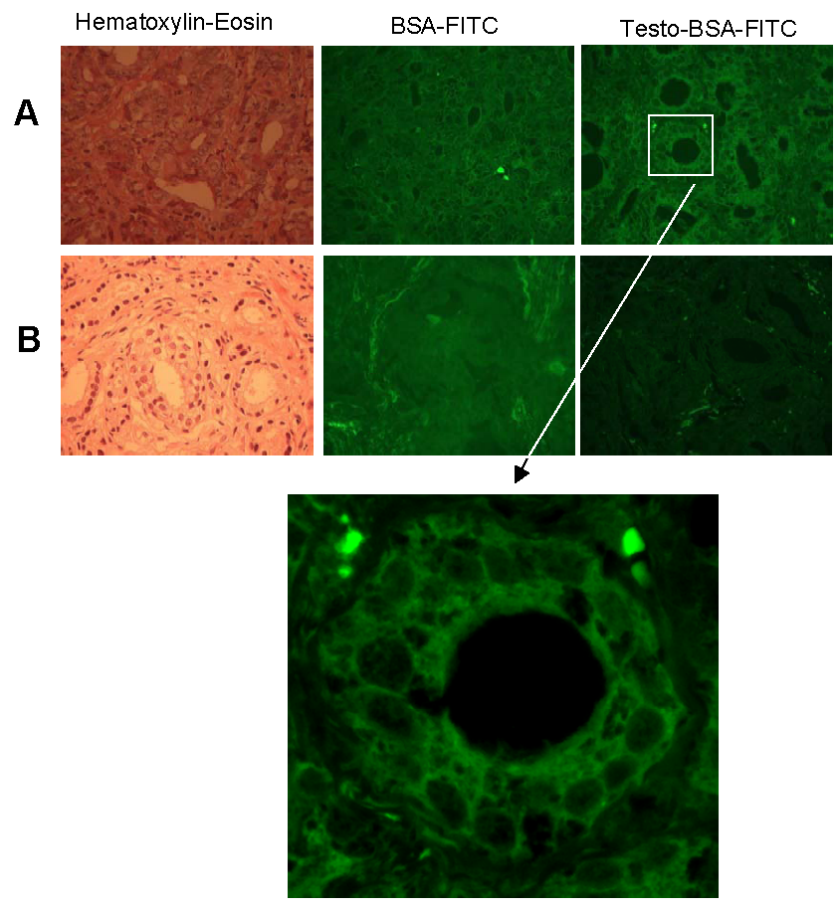
*Representative cases of mAR positive (A) and negative (B) tumor specimens*. A representative case of mAR positive (A) and a negative case (B) are shown. Left panels present H&E staining, middle panels present BSA-FITC staining, while right panels present staining with testosterone-BSA-FITC. Note the membrane localization of fluorescence in positive cells. Lower panel presents a higher magnification of the square region shown. In all cases preincubation with 3% BSA and 10^-4^M cyproterone acetate was performed, prior to mAR detection. Compare Figure 2 with Figure 1 (no preincubation).

When slides were incubated with testosterone-BSA-FITC without any treatment, they presented a high non-specific binding (Figure 1, upper panel), due probably to the non-specific absorption of BSA to membrane and/or intracellular structures or molecules. For this reason, slides were preincubated with a 3% BSA solution prior to ligand binding. As shown (Figure 2A and 2B) this treatment resulted in an attenuation of conjugated or unconjugated BSA-FITC to membranes.

Another matter of concern was that iARs, regularly present in prostate carcinoma cells, could bind the ligand since, in tissue sections, one expects an interaction of the conjugated ligand with membrane and (due to sectioning) intracellular structures and/or molecules, including iARs. Our previous results with prostate cancer cell lines have shown that interaction of testosterone-BSA with mARs is not influenced (at the level of binding or the resulting signaling cascade and secretion) by antiandrogens [6,9] Therefore, to eliminate a possible interaction of BSA conjugated ligand with iARs, we used 10^-4^M cyproterone acetate in the binding experiments.

This treatment decreased nuclear, membrane and intracellular staining (Figure 1). The treatment of slides with testosterone-BSA-FITC in the presence of 3% BSA and 10^-4^M cyproterone acetate decreased non-specific staining in nearly background levels, making more discernible the interaction of testosterone-BSA-FITC with specific membrane-binding sites (compare Figure 1 with Figure 2A right panel).

According to the above results, for the detection of mARs in tissue sections, we applied BSA (3%) and cyproterone acetate (10^-4^M) throughout the present study. These conditions were found to accurately discriminate between positive (>75% of cells labeled by testosterone-BSA-FITC) and negative (<10% of cells labeled by testosterone-BSA-FITC) cases.

### 2. Prostate carcinoma specimens preferentially express mARs

On examination of the specimens, positive cases of both carcinomas and BPHs were found to present peripheral, membrane type, fluorescence. In contrast, morphologically normal-looking prostate epithelial tissue showed no specific fluorescence. In Figure 2, representative cases of prostate carcinoma are presented, showing a positive (Figure 2A) or negative (Figure 2B) staining for mARs. Positive cases showed peripheral, continuous and/or stippled, membrane type, fluorescence. This extended over more than 75% of carcinoma cells of the tissue section. Negative cases, on the other hand, showed no more than 10% reacting cells. Intensity of staining in positive cells was not considerably different among cases. Thus, reading and translating mAR positivity seems to be a matter of nearly a "black and white" effect and membrane pattern recognition.

**Figure 3:**
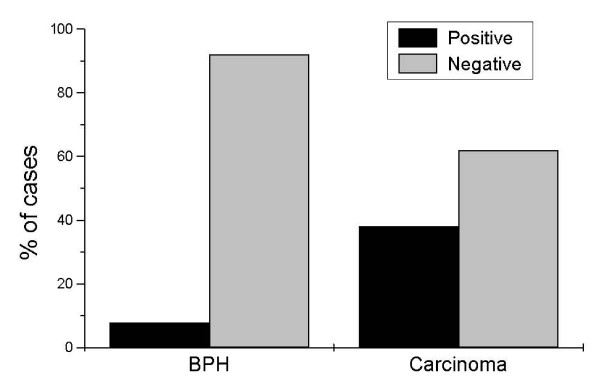
*Distribution of positive and negative mARs in BPH and prostate carcinoma*. Ninety % of benign prostate hyperplasia cases (n = 103) were negative for mAR receptor expression. On the other hand, prostate carcinoma cases (n = 106) showed mAR expression in a significant percentage (38% of these cases were positive) (χ^2 ^= 16.7, p < 0.0001).

The tissue stroma sometimes showed a diffuse positive reaction, probably due to connective tissue non-specific binding. This non-specific staining did not interfere, though, with the estimation of mAR expression in epithelial cells of the tissue section. Ninety percent (90%) of BPHs were negative for mAR, while about 38% of carcinomas were positive (Figure 3) (χ^2 ^= 23.24, p < 0.0001). No correlation was found between mAR expression and age of the patient, treatment before or after surgery and survival.

### 3. Membrane Androgen Receptor sites are related to the level of differentiation of prostate carcinoma

**Figure 4:**
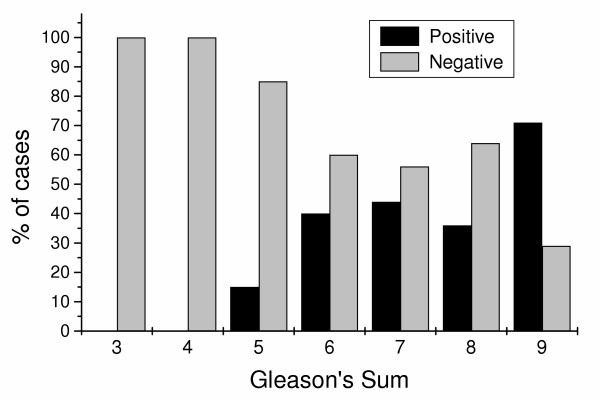
*Correlation of mAR positivity with Gleason's sum in prostate carcinomas*. Membrane androgen receptors are preferentially expressed in prostate carcinomas and their presence is correlated to Gleason's sum.

As mentioned above, 38% of prostate carcinomas were found to express mARs (χ^2 ^6.68, p < 0.1, among all cases of carcinoma, n = 109). Thus, mAR expression cannot be used for the discrimination between malignant and benign prostate cases. To evaluate the possible role of mARs in the evolution of prostate carcinoma, we correlated the presence or absence of mARs to the differentiation of the prostate carcinoma, estimating the Gleason's sum of each case on the corresponding H&E stained slide. Using correlation analysis, our results showed that mAR expression is positively related to higher Gleason's sum (Mann-Whitney U = 991, p < 0.04, Spearman's rho = 0.236, p < 0.01, Figure 4). It is worthy to note that 50% of poorly differentiated carcinomas (Gleason's sum 8 and 9, in our series) expressed mARs while in the Gleason's sum 9 carcinomas, mARs expression predominated. As seen in Figure 4, there is an increased expression of mARs with increasing Gleason's sum (15%, 40%, 44%, 36%, 71%, for Gleason's sums from 5 to 9 respectively). No difference was found between Gleason's sum 4+3 and Gleason's sum 3+4 regarding mAR expression (11 out of 21 cases of Gleason's sum 3+4, and 9 out of 20 cases of Gleason's sum 4+3 expressed mAR).

Different percentages of mAR positivity were revealed when prostate carcinoma cases were stratified according to the type of tissue sample. The number and percentages of positive carcinoma cases in each type of specimen is presented in Table 2. Radical prostatectomies showed 13.04% positivity and transurethral resection specimens showed 26.22%, while almost half of the transrectal biopsies were found to express mARs (47.80%). The number of transrectal biopsies (68 studied) was higher correlated to the number of cases of the other two groups (radical prostatectomies 23 and transurethral resections 15). Statistical analysis showed that there is a significant difference in mAR expression between the three types of tissue samples (binomial distribution comparison, p < 0.01).

## Discussion

The classical mode of action of steroid hormones includes their entrance into hormone-dependent cell and binding to their intracellular cognitive receptors, which subsequently dimerize and translocate to the nucleus, acting as specific transcription factors [14]. This sequence of events requires time for their completion (>2 hours). In recent years, however, steroids were found to exert a number of effects in seconds or minutes, a phenomenon not explained by their intracellular action. Ion mobilization, activation of specific signaling cascades or secretion are included among these rapid actions and are considered non-genomic, independent of transcription or translation [[15], see [16], for a review]. A number of possible explanations for the non-genomic steroid actions include the presence of membrane binding sites, different from intracellular receptors [17], the anchorage of intracellular receptors to the plasma membrane through post-translational modification of receptor molecules [18], the interaction of intracellular steroid receptors with other membrane-bound receptors, or the activation of signaling molecules through intracellular receptor-bound steroids.

Recently, a progesterone binding membrane site has been isolated and found to belong to the seven-loop G-protein coupled receptor superfamily [19]. Data on other steroids binding membrane sites have also been reported [2,4,20-22], although their molecular nature has not been elucidated until now. More recently, we have reported the identification of mARs in prostate cancer cell lines [5,6] and in a small series of human prostate carcinomas [8]. Membrane androgen receptor sites activation induces Ca^2+ ^flux [23], actin reorganization [9] and PSA secretion [5], in an antiandrogen-independent manner, inducing apoptosis in vitro [6,7] and in vivo [6]. In addition, tritiated testosterone has been found to bind efficiently and specifically to acid-stripped membranes [5].

The above data indicate that the detection of mARs could be of value in the diagnosis; and possibly mARs themselves could be exploitable in a new therapeutic approach of the mAR positive prostate carcinomas, as prostate carcinoma xenografts in nude mice are significantly reduced in size by testosterone-BSA treatment of animals [6]. In the present study we investigated whether mARs could also be detected in formalin-fixed and paraffin-embedded specimens of prostate carcinoma. The main disadvantage in mARs detection is that, until the elucidation of their molecular structure, no antibodies interacting with them are available. In addition, antibodies to iAR do not cross-react with mAR [7]. Therefore, for the time being, the only appropriate method for mAR detection is that based on the use of labeled non permeable ligands, such as testosterone-BSA-FITC. Previous results of experiments of our team performed in formalin- or PFA-fixed cell lines (LNCaP, DU-145, and PC12) and circulating leukemic lymphocytes have shown that mAR could be effectively detected on the cell surface. In view of these data, in the present study we used mild deparaffinization, in combination with citrate buffer pretreatment, known to induce dissociation of ligands or iAR loosely-bound on membrane sites [7,13]. Our results indicate that, under such conditions, mARs could be detected in prostate carcinomas on formalin-fixed and paraffin embedded tissue sections.

A problem encountered in our attempt to reveal *in situ *mARs was that BSA-FITC bound non-specifically to/or it was absorbed by stromal structures or cell and/or intracellular components. In order to decrease this non-specific interaction, we pre-incubated tissue sections on slides with high concentrations of BSA, thus eliminating or efficiently decreasing this interaction. Intracellular ARs (iARs) do not interfere with the detection of mARs by the method presented here. Indeed, as found in our previous studies, these latter sites do not react with antiandrogens, which can block intracellular receptors [6,9]. We have therefore exploited this differential antiandrogen selectivity in order to block selectively iAR binding, by the use of high concentrations of the antiandrogen cyproterone acetate. In addition, we report that, although confocal microscopy could be considered more precise and accurate than the -cheaper in hardware- conventional fluorescence microscopy, once the researcher becomes accustomed to mAR expression pattern, conventional fluorescence microscopy can be a valuable alternative in revealing the presence of mAR in tissue sections.

Our findings indicate that intensity is not a factor to take into account in the evaluation of mAR expression, since, in individual positive cases, a rather uniform staining intensity was observed. In the case of advanced prostate carcinomas there is a heterogeneity of cell populations expressing iAR [24,25], suggesting iAR counting in hot spots. Such heterogeneity was not observed in our study of mARs, and thus positivity was estimated in 10 sequential high power fields in representative tissue section regions (X400).

The most appropriate samples for the study of mAR expression seem to be the transrectal biopsies (50% positivity), which constituted most of our specimens (71 out of 106 finally studied), followed by the TUR specimens (26.66% positivity), and the radical prostatectomy specimens (13.04% positivity). This signifies that the quality of fixation (as depended on time and room temperature) is of critical importance in the study of mARs.

We report that only 38% of prostate carcinoma cases are positive for mAR staining. In this respect, mAR positivity could not be a discriminant factor for prostate cancer diagnosis. Stratification of cases, according to the Gleason's sum revealed that mAR-positive staining correlates with less differentiated tumors. No significant difference in mAR expression between Gleason's sum 4+3 (9 out of 20 cases) and Gleason's sum 3+4 (11 out of 21 cases) prostate carcinomas was found. Nevertheless, no solid conclusions can be drawn, due to the relatively small number of cases studied.

Membrane androgen receptors are not only expressed in the prostate. They have been found in osteoblasts, lymphoid cells and Sertoli cells [reviewed in [12]] independently of the presence or not of iARs. Preliminary data from our group studies indicate that mARs are expressed in higher grade carcinomas of the breast, B-lymphomas, pheochromocytomas and in AC133^+ ^cord blood stem cells (unpublished observations). In addition, in estrogen receptor negative (ER-negative) carcinomas of the breast we have found a strong staining for mAR [7]. In this respect, mAR might be considered rather as a marker of "immature" and behaviorally aggressive cells than a tissue specific marker. If this comes true, it could explain the finding that tumors with high Gleason's sum preferentially express mARs.

## Conclusion

Our results indicate that mARs detection cannot discriminate between prostate carcinoma and BPH, not being exploitable in the differentiation between low-grade carcinomas and BPHs. Nevertheless mARs could be used for a biologic grading of prostate carcinomas, since the more aggressive ones-of higher histopathological grade- preferentially express mARs. Results of recent studies of our team indicate that agonists of mARs-being expressed in cases of both iAR-positive and iAR-negative prostate cancer cell lines-might act as specific therapeutics directing carcinoma cells towards apoptosis [6]. In addition, the activation of mAR-signaling cascade leads to apoptosis [6], while activation of iARs is antiapoptotic [26]. This, in combination with the finding that prostate carcinomas of low Gleason score have a significantly higher iAR content than those with high Gleason score [27], signifies a different biological role for mARs in relation to iARs. Hence, the possibility that combined examination of iARs and mARs in prostate carcinomas, in the context of a test clinically applicable and easy to perform, could lead to a more accurate biologic grading of predictive and/or prognostic importance. To test the prognostic power of mARs, further studies in prostate carcinomas are needed. Moreover, mARs' study in large series of prostate carcinomas is imperative in order to fully elucidate the pathway of their action and reveal whether they represent a possible target for new therapies of these carcinomas, especially in view of our results on regression of prostate tumor xenografts by testosterone-BSA [6].

## Competing interests

The author(s) declare that they have no competing interests.

## Authors' contributions

CD, CK, MK and KD have performed the collection, staining and evaluation of cases; MN and PA collected the clinical data, PAT, EC and ENS supervised and controlled the whole study

## Pre-publication history

The pre-publication history for this paper can be accessed here:



## Figures and Tables

**Table 1:** Type of specimens and their corresponding Gleason's sum.

**Type of specimen**	**Gleason's sum**										
		
	**2**	**3**	**4**	**5**	**6**	**7**		**8**	**9**	**10**	**Total number**
										
						**3+4**	**4+3**				
**Radical prostatectomy**	2	0	2	2	7	2	4	2	2	0	23
**Transurethral resection**	2	0	1	5	0	2	0	2	3	0	15
**Transrectal biopsy**	1	0	3	3	18	17	16	9	1	0	68
**Total**	5	0	6	10	25	21	20	13	6	0	106

**Table 2:** Type of specimens and results of the study for mAR expression

**Type of specimen**	Positive	Negative	Rejected	Total	% of positivity
**Radical prostatectomy**	3	20	0	23	13,04
**Transurethral resection**	4	11	0	15	26,66
**Transrectal biopsy**	34	34	3	71	50,00
**Total**	41	65	3	109	38,60
